# Advances in MXene-Based Electrochemical Sensors for Multiplexed Detection in Biofluids

**DOI:** 10.3390/ijms26115368

**Published:** 2025-06-03

**Authors:** Meiqing Yang, Congkai Xie, Haozi Lu

**Affiliations:** 1 Zoology Key Laboratory of Hunan Higher Education, College of Life and Environmental Science, Hunan University of Arts and Science, Changde 415000, China; 2 State Key Laboratory of Chemo/Biosensing and Chemometrics, College of Chemistry and Chemical Engineering, Hunan University, Changsha 410082, China; xiecongkai@hnu.edu.cn (C.X.); luhaozi@hnu.edu.cn (H.L.)

**Keywords:** electrochemical sensors, multiplexed, simultaneous, biofluids, MXene

## Abstract

Detection of multiple analytes in biofluids is of significance for early disease diagnosis, effective treatment monitoring, and accurate prognostic assessment. Electrochemical sensors have emerged as a promising tool for the multiplexed detection of biofluids due to their low cost, high sensitivity, and rapid response. Two-dimensional transition metal carbon/nitride MXene, which has the advantages of a large specific surface area, good electrical conductivity, and abundant surface functional groups, has received increasing attention in the electrochemical sensing field. This paper systematically reviews the advances of MXene-based electrochemical sensors for multiplexed detection in biofluids, emphasizing the design of MXene-based electrode materials as well as the strategies for distinguishing multiple signals during simultaneous electrochemical analysis. In addition, this paper critically analyzes the existing challenges of MXene-based electrochemical sensors for multiplexed detection of biofluids and proposes future development directions for this field. The ultimate goal is to improve biofluid multiplexed detection technology for clinical medical applications.

## 1. Introduction

Biofluids (e.g., blood, urine, sweat, tears, saliva, interstitial fluid, cerebrospinal fluid, etc.) are an important part of living organisms, which contain a wide range of components such as ions, chemical molecules, biological metabolites, proteins, nucleic acids, and even substances with cellular structure [1,2]. Generally, these components vary within a certain concentration range and their abnormal levels often reflect certain health problems. Therefore, the biofluid detection is crucial for health maintenance, disease management, and personalized treatment [2]. In fact, biofluid detection has become an indispensable part of modern medicine [3]. Conventional analytical techniques, such as nuclear magnetic resonance (NMR), mass spectrometry (MS), liquid chromatography/gas chromatography (LC/GC), liquid chromatography–mass spectrometry (LC-MS) and gas chromatography–mass spectrometry (GC-MS), have been commonly used for clinical biofluid analysis [4,5]. Nonetheless, these conventional techniques are limited by complex and time-consuming procedures, expensive equipment, or trained technicians, and a requirement for fixed laboratory settings. In contrast, electrochemical sensing has emerged as a promising alternative due to its cost-effectiveness, simplicity, high sensitivity and selectivity, rapid response, and potential for miniaturization [6]. More importantly, it enables on-site detection and real-time monitoring [7]. Compared to single-target detection, simultaneous detection of multiple analytes presents significant advantages of reduced sample volume, lower cost, and shorter analysis time [8]. In particular, multiplexed detection is more accurate in disease diagnosis by providing more comprehensive feedback [9]. Hence, the multiplexed electrochemical sensors are receiving increasing attention.

Electrodes, as sensing interfaces, play a crucial role in electrochemical sensing. Bare electrodes usually exhibit low sensitivity and selectivity towards analytes owing to sluggish surface dynamics [10]. Moreover, it is challenging for bare electrodes to discriminate multiple analytes [11]. To address these challenges, considerable efforts have been devoted to selecting appropriate electrode modification materials to improve the performance of electrochemical sensors. The application of various nanomaterials, such as metal nanoparticles (e.g., AuNPs, PtNPs), metal oxide nanomaterials, quantum dots, carbon nanotube/nanofibers, graphene and its derivatives, conducting polymers, metal organic frameworks, ionic liquids, transition metal dichalcogenides, MXene, etc., has significantly advanced the development of electrochemical sensors [12,13,14,15,16]. Among them, the unique physicochemical properties of MXene enable it to overcome the challenges faced by other nanomaterials in electrochemical sensing, including hydrophobicity, insufficient electrical conductivity, difficulty in surface functionalization, and limited biocompatibility, making it a highly promising material for constructing electrochemical sensors. Specifically, as a two-dimensional (2D) layered material, MXene has a large specific surface area (up to 235.6 m^2^/g) [17], which can provide more surface-active sites. Unlike other nanomaterials, MXene possesses abundant surface functional groups and can be regulated through different preparation methods and post-treatment strategies, making it easy for surface modification and functionalization. Furthermore, compared with other 2D materials (e.g., hydrophobic graphene and poorly conductive transition metal dichalcogenides), MXene has both high electrical conductivity (up to 2.4 × 10^4^ S/cm) [18,19] and good hydrophilicity, which makes it more suitable for the preparation of high-performance electrochemical sensors. Finally, MXene also has good biocompatibility [20], which is essential for the reliability of electrochemical biosensors.

To date, numerous review articles have addressed the application of MXene in electrochemical sensing, which summarized its utility in the detection of environmental pollutants, food safety, biomarkers, and pathogens [21,22,23,24,25,26,27,28,29,30,31]. With the growing public demand for multiplexed detection, MXene-based electrochemical sensors for multiplexed detection have been increasingly reported since 2017. However, a systematic summary detailing the application and design strategies of MXene-based multiplexed electrochemical sensing is still lacking.

This paper systematically summarizes the application of MXene-based multiplexed electrochemical sensors for biofluid detection, emphasizing the design and preparation of MXene-based electrode materials as well as the strategies for distinguishing between different target signals. In addition, the current problems of MXene-based multiplexed electrochemical sensors for biofluid detection are analyzed, and practical recommendations for future advancements in this field are presented. The aim of this paper is to promote MXene-based multiplexed electrochemical sensors for the benefit of human health business as soon as possible.

## 2. Brief Introduction to MXene

**Figure 1:**
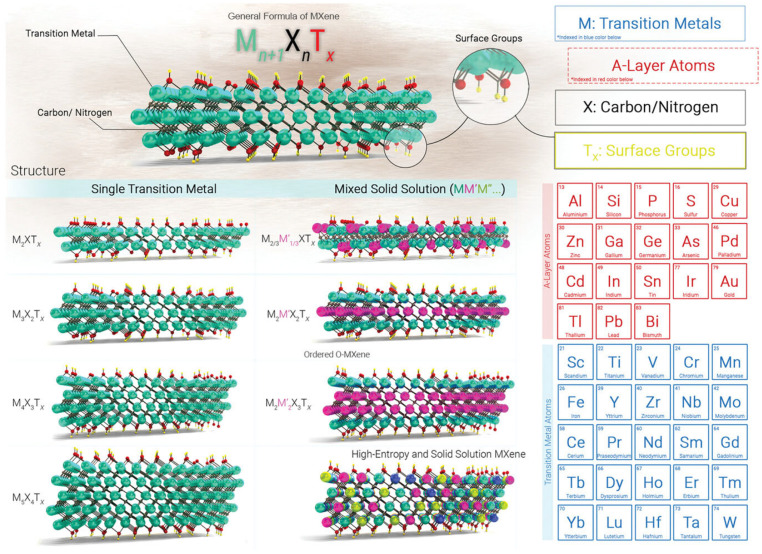
Schematic of the different molecular structures of MXene and the constituent elements of MAX and MXene. Reproduced with permission from [36]. * (blue) refers to the elements in blue color below (Sc, Ti, V, etc.), and * (red) refers to the elements in red color below (Al, Si, P, etc.).

Since the initial discovery of Ti_3_C_2_T_x_ in 2011 [32], MXene has evolved into a large family of 2D transition metal carbides, nitrides, and carbonitrides. To date, more than 45 MXene species have been experimentally synthesized [33]. MXene can be represented by the formula M*_n+_*_1_X*_n_*T*_x_*, where M stands for the transition metal, X denotes carbon (C) and/or nitrogen (N), T*_x_* represents the surface functional groups (e.g., -OH, -O, -F, and -Cl), and *n* is 1, 2, 3, or 4 [34,35]. Based on the value of *n*, the molecular structure of MXene can be classified into four categories: M_2_XT_x_, M_3_X_2_T_x_, M_4_X_3_T_x_, and M_5_C_4_T_x_ [36]. Typically, MXene is prepared by etching the A atomic layer from its parent MAX phase, in which A mainly corresponds to the elements of IIIA-VIA groups. Figure 1 shows the different structures of MXene and the constituent elements of MAX and MXene.

MXene was first produced using the HF etching method. Recognizing the hazards associated with HF, alternative approaches such as in situ HF etching (acid/fluoride salt etching) [37] and NH_4_HF_2_ etching [38] were later proposed. Subsequently, some fluorine-free preparation techniques aimed at eliminating fluorine residues have been reported, including electrochemical etching [39], alkali etching [40], molten salt etching [41], and halogen etching [42]. All of the above methods for obtaining MXene through MAX precursor etching are categorized as “top-down” methods. Furthermore, MXene can also be synthesized through “bottom-up” methods, such as chemical vapor deposition (CVD) [43], plasma-enhanced pulsed laser deposition (PEPLD) [44], template-assisted growth [45], and magnetron sputtering [46]. The “top-down” methods are currently predominant in MXene preparation, but they inevitably introduce surface functional groups and defects. In contrast, “bottom-up” methods are more attractive as they hold promise for achieving higher crystallinity [33,47], but they also suffer from the problems of expensive equipment, harsh conditions, or complicated procedures. Therefore, exploring green, simple, and efficient MXene preparation techniques is still underway. MXene obtained by the etching process exhibits an accordion-like multilayered structure, with adjacent nanosheets interconnected by hydrogen bonding and van der Waals forces. Compared to multilayered MXene, single- or few-layered MXene exhibits enhanced performance in surface area, hydrophilicity, and electrochemical activity [47]. Therefore, the etched MXene usually requires some intercalators for delamination. Specifically, certain metal cations (e.g., Li^+^, Na^+^, K^+^), organic compounds (urea, hydrazine, dimethylsulfoxide (DMSO), isopropyl amine (i-PrA), n-butyl amine (n-BA), tetrabutylammonium hydroxide (TBAOH), tetramethylammonium hydroxide (TMAOH), and choline hydroxide (ChOH)), or polymer molecules (polyvinyl alcohol) can be intercalated into the interlayer of MXene to expand spacing [21,48,49], and then the delamination of MXene can be forced by mechanical methods (such as sonication). It is noteworthy that in situ HF etching does not require intercalation, as the cations in the fluoride salts enable simultaneous etching and intercalation [47].

## 3. Strategies for Discriminating Different Target Signals in Simultaneous Electrochemical Detection

The primary challenge in simultaneous electrochemical detection is how to discriminate signals from different analytes [50,51]. Depending on the analyte, simultaneous electrochemical detection can be achieved by different strategies. For electrochemically active analytes with sufficiently different redox potentials, simultaneous detection can be performed directly. Even so, enhanced detection performance (e.g., higher sensitivity and lower detection limit) can be achieved by appropriate modification of the electrode. For electrochemically active analytes with similar redox potentials, electrode modification becomes essential to alter the kinetics of the redox reaction and, in turn, differentiate the analytes [52,53]. Typically, dopamine (DA), ascorbic acid (AA), and uric acid (UA) coexist in biofluids and show overlapping oxidation potentials on bare electrodes. By modifying the electrodes with superior electrochemical nanomaterials, the oxidation peaks of these biomolecules can be effectively separated [54]. Another feasible strategy to identify electrochemically active analytes with similar redox potential is to design multiple working electrodes. For instance, a paper-based analytical device utilizing two separate working electrodes along with a shared reference and counter electrode was developed for the simultaneous electrochemical detection of norepinephrine and serotonin [55].

**Figure 2:**
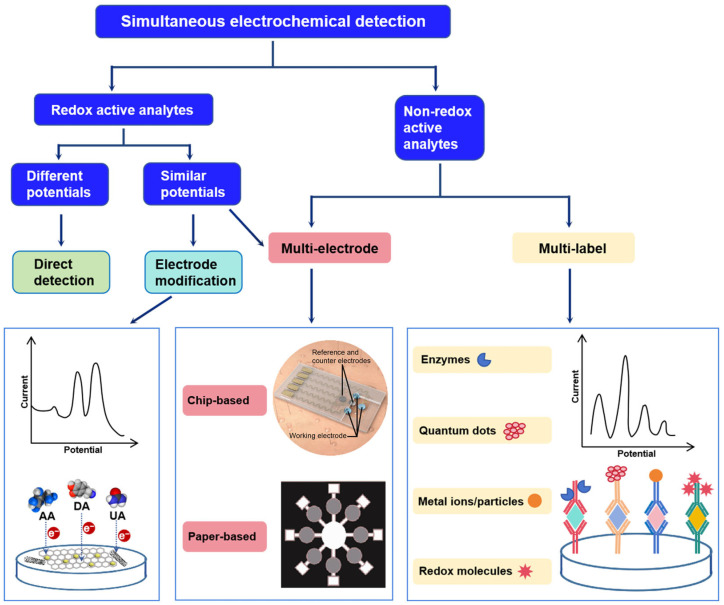
Schematic diagram for simultaneous electrochemical detection.

For analytes that lack electrochemically activity (e.g., proteins, nucleic acids, cell), a multi-electrode or multi-label strategy can be employed to distinguish between targets [50,56,57,58]. Multi-electrode platforms mainly include chip-based and paper-based electrode arrays [8]. The multi-electrode strategy has the advantage of robustness, user-friendliness, and high sensitivity and accuracy [50,57]. However, a multi-electrode system usually requires a multi-channel electrochemical analyzer (e.g., multi-channel potentiostat), which leads to a relatively high detection cost [53]. The multi-label strategy enables simultaneous detection in a single run on one working electrode. To date, various types of labels including enzymes, redox active organic molecules, Prussian blue (PB), metal nanoparticles, metal ions, quantum dots, magnetic beads, etc., have been employed for the simultaneous detection of multiple analytes [50,52,59,60]. Horseradish peroxidase (HRP), alkaline phosphatase (ALP), laccase, and glucose oxidase (GOx) are the commonly used enzyme labels. The use of enzyme labels requires the addition of specific substrates to the testing solution [61]. Redox active organic molecules include methylene blue (MB), thionine (Thi), ferrocene (Fc), toluidine blue (TB), neutral red (NR), anthraquinone 2-carboxylic acid (AQ), etc. [59,62]. Multi-label strategy using a single electrode is characterized by its simplicity and cost-effectiveness, but it faces the challenge of electrochemical signals interfering with each other (“cross-reactivity”), which needs to be solved by selecting appropriate redox active labels [61]. Notably, multi-electrode strategy was sometimes coupled with multi-label strategy to achieve target differentiation [63,64]. A summary of the strategies for simultaneous electrochemical detection is illustrated in Figure 2, and a comparative table illustrating the advantages and disadvantages of these strategies is demonstrated in Table 1.

## 4. MXene-Based Electrochemical Sensors for Multiplexed Detection in Biofluids

To date, a certain number of MXene-based electrochemical sensors have been developed for multiplexed detection of various analytes in biofluids. These sensors are mainly utilized for the detection of heavy metal ions, biomarkers (electrolytes, metabolites, neurotransmitters, proteins, and nucleic acids) as well as drugs in biofluids such as sweat, urine, serum, plasma, whole blood, and saliva. Various analytical methods are employed for analyte detection, including differential pulse voltammetry (DPV), square wave voltammetry (SWV), square wave anodic stripping voltammetry (SWASV), chronoamperometry (CA), amperometry, cyclic voltammetry (CV), and potentiometry. Table 2 provides an overview of these MXene-based electrochemical sensors, detailing working electrodes, signal separation strategies, target analytes, involved biofluids, analytical methods, and performance. Some representative MXene-based electrochemical sensors for multiplexed detection in biofluids are described below.

### 4.1. Heavy Metal Ions

Heavy metal pollution currently poses a significant threat to human health. These toxic metal ions can bioaccumulate and eventually enter the human body along the food chain, leading to diseases of the heart, kidneys, liver, central nervous system, and reproductive system. Some of these heavy metals, such as Cu^2+^ and Zn^2+^, are essential trace elements for the human body that play an important role in maintaining physiological functions, but their excess or deficiency can also result in serious health problems. In addition, the synergistic effect and additive toxicity of multiple heavy metal ions are more pronounced than that of a single metal [92]. Therefore, the simultaneous detection of heavy metal ions within the human body is crucial for mitigating health risks.

Anodic stripping voltammetry (ASV) is the predominant analytical method used for trace detection of metal ions, consisting of two steps: deposition and stripping. In the deposition step, the target ions are reduced onto the electrode surface. Therefore, it is particularly important to select electrode materials that can effectively “attract” the target ions. In the past, mercury (Hg) was the primary material utilized for heavy metal ion detection due to its ability to form metallic alloys with various ions. However, its inherent toxicity has restricted its widespread application. Recent research efforts have focused on identifying environmentally friendly alternatives to Hg.

**Figure 3:**
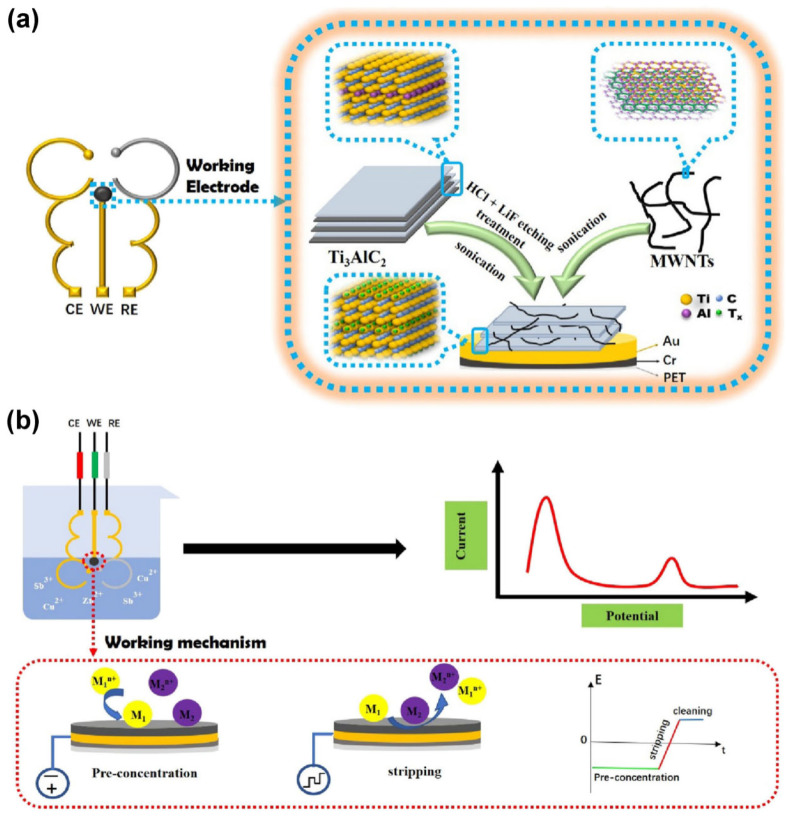
Fabrication process of flexible Ti_3_C_2_T_x_/MWCNTs/Au electrode (**a**) and its working mechanism for Cu^2+^ and Zn^2+^ detection (**b**). Reproduced with permission from [65].

Hui et al. [65] prepared a flexible electrochemical sensor based on a gold (Au) electrode modified by the layer-by-layer assembly of Ti_3_C_2_T_x_ and multiwalled carbon nanotubes (MWCNTs) nanocomposites for the non-invasive detection of Cu^2+^ and Zn^2+^ (Figure 3). The introduced MWCNTs not only alleviate the stacking problem of Ti_3_C_2_T_x_ nanosheets, but they also synergize with Ti_3_C_2_T_x_ to improve their mutual electrochemical properties. Moreover, an environmentally friendly mercury alternative, antimony (Sb), was electrodeposited in situ to further enhance analytical performance. Using SWASV measurements, the prepared sensor exhibited good analytical performance with a wide detection range (Cu^2+^: 10–600 ppb, Zn^2+^: 350–830 ppb) and low detection limits (Cu^2+^: 0.1 ppb, Zn^2+^: 1.5 ppb), and has been successfully employed for the detection Cu^2+^ and Zn^2+^ in both sweat and urine.

### 4.2. Biomarkers

Biomarkers, such as electrolytes (Na^+^, K^+^), small molecules (metabolites, most neurotransmitters), and macromolecules (proteins, nucleic acids), are closely related to information about the body’s physiological state [77,93,94]. Electrolytes are necessary for the regulation of body functions such as nerve transmission, hormone secretion, muscle contraction, acid–base balance, enzyme activation, and blood pressure control [95]. Metabolite levels are important for precise screening, monitoring, and prognosis of metabolic disorders and relevant diseases [96]. Neurotransmitters are important biomarkers for various neurological disorders, such as Parkinson’s disease, Alzheimer’s disease, and depression [97]. Proteins and nucleic acids are associated with a wide range of diseases such as cancer and infectious diseases [8]. Hence, the detection of biomarkers is of significance in diagnosing diseases and monitoring the health status of individuals.

#### 4.2.1. Small Molecules

Dopamine (DA), uric acid (UA), and ascorbic acid (AA) are always coexisting in the extracellular fluids of the central nervous system and serum in humans [98]. DA is a neurotransmitter in the central nervous system that plays an important role in message transmission. Its low levels can lead to brain disorders such as schizophrenia and Parkinson’s disease. UA is a major final product of purine metabolism, and its abnormal levels are associated with gout and other related complications. AA (also known as vitamin C) is a vital vitamin in the human diet. Its deficiency can lead to gum bleeding and scurvy, while its excessive intake can cause diarrhea, urinary stones, and stomach cramps [99]. However, the simultaneous electrochemical detection of DA and UA in biofluids is challenging because coexisting high concentrations of AA interfere with DA and UA, and the oxidation potentials of AA overlap with those of DA and UA [98]. To address this challenge, different electrode modifications have been proposed.

Murugan et al. [66] successfully prepared a 2D Ti-C-*T_x_*-MXene mixed-phase by a facile one-step synthesis and modified it on a glassy carbon electrode (GCE) to obtain Ti-C-*T_x_*/GCE. The obtained Ti-C-*T_x_*/GCE exhibited excellent electrocatalytic activity and good selectivity for AA, DA, and UA. Moreover, the Ti-C-T_x_/GCE achieved satisfactory results in the detection of AA, DA, and UA in human urine samples. In one study, a flexible MXene-based electrochemical sensor was developed for the simultaneous detection of AA, DA, and UA [67]. To prepare the sensor, three-dimensional (3D) laser-scribed porous graphene (LSG) was first functionalized with Ti_3_C_2_T_x_ MXene via a C-O-Ti covalent crosslink to form an LSG-MXene hybrid scaffold, and then Au-Pd bimetallic nanoparticles were self-reduced on the surface of the scaffold to enhance the catalytic property. The obtained Au-Pd/MXene/LSG sensor with high sensitivity was successfully applied for the simultaneous detection of AA, DA, and UA in urine samples. In another study, Jia and colleagues synthesized a composite of TiO_2_ nanowires grown in situ on a Ti_3_C_2_T_x_ substrate (Ti_3_C_2_T_x_/TiO_2_ NWs) by a simple alkaline process [68]. These TiO_2_ nanostructures not only alleviated the aggregation of Ti_3_C_2_T_x_ nanosheets, but they also increased the electrocatalytic activity of the MXene-based composites. By modifying GCE with Ti_3_C_2_T_x_/TiO_2_ NWs, the obtained modified electrodes showed excellent performance in the simultaneous detection of AA, DA, and UA with low detection limits (AA: 6.61 μM, DA: 0.093 μM, UA: 0.038 μM).

**Figure 4:**
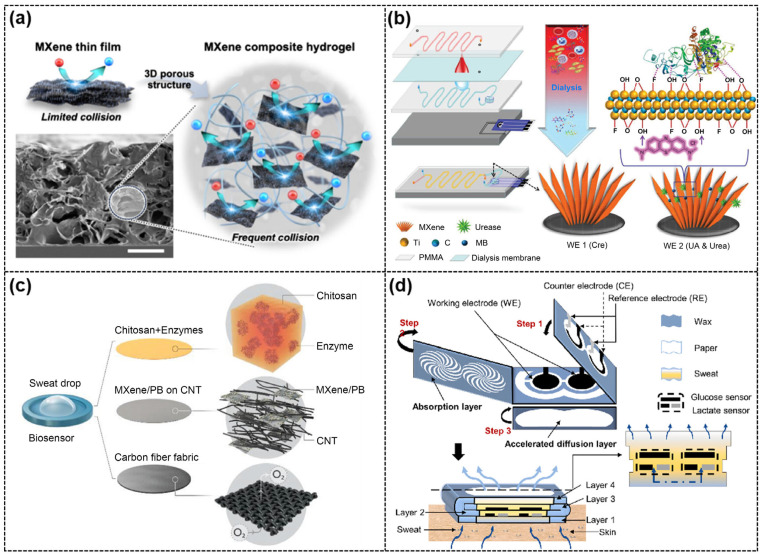
(**a**) Schematic of 2D MXene structure (**top left),** 3D MXene composite hydrogel (**bottom right**), and SEM image of the 3D MXene composite hydrogel (**bottom left**, scale bar = 10 μm). Reproduced with permission from [71]. (**b**) Schematic of the fabrication of a MXene-based microfluidic chip for the simultaneous and continuous analysis of urea, UA, and Cre in whole blood. Reproduced with permission from [74]. (**c**) Schematic diagram of oxygen-enriched GOx(LOx)/CNTs/Ti_3_C_2_T_x_/PB/CFM electrode. Reproduced with permission from [76]. (**d**) Structural diagram of the HIS paper. Reproduced with permission from [77].

Simultaneous monitoring of neurotransmitters and antioxidants is crucial for gaining valuable insights into the initiation and progression of various neurological disorders. The unique properties of MXene make it suitable for integration into hydrogel-based sensors. To this end, researchers introduced MXene into poly(ethylene glycol) diacrylate (PEGDA) to prepare a silane-functionalized MXene-PEGDA hydrogel for the simultaneous detection of DA, 5-hydroxytryptamine (5-HT) (neurotransmitters), and UA (antioxidant) [71]. Specifically, MXene was first functionalized with γ-KH570 to form acrylate-terminated MXene (Ac-MX), and then Ac-MX was mixed with PEGDA, a photoinitiator, and CaCl_2_ for ionic cross-linking under UV light to obtain the 3D porous MXene-PEGDA hydrogel. It is worth mentioning that the functionalization of MXene with silane molecules improved its activity and stability in the PEGDA hydrogel matrix. The specific surface area of the synthesized 3D composite hydrogel was significantly increased compared to 2D MXene, providing more collision frequencies for the target molecules (Figure 4a). As a result, the composite hydrogel exhibited excellent electrooxidation of DA, 5-HT, and UA, and achieved a wide linear detection range (DA: 2.5–200 μM, 5-HT: 1–100 μM, UA: 10–100 μM). More importantly, the silane-functionalized MXene-PEGDA hydrogel showed good selectivity for the simultaneous detection of DA, 5-HT, and UA in complex human serum samples.

Folic acid (FA) is an important water-soluble vitamin B for humans, and its deficiency can lead to health problems such as cardiovascular disease, cancers and neural tube defects in newborns [100]. In addition, the oxidation potential of FA overlaps with that of UA, hindering their simultaneous detection. Elumalai et al. [72] prepared AuNP@Ti_3_C_2_T_x_ composite films by spontaneously reducing AuNP on the delaminated Ti_3_C_2_T_x_ nanosheets, which were then modified on GCE for simultaneous detection of UA and FA. Thanks to the good synergy between Ti_3_C_2_T_x_ nanosheets and AuNP, the oxidation peak potentials of UA and AA were well separated (+0.35 V for UA and +0.70 V for FA). Adenine and guanine are indispensable components of nucleic acids, and their metabolic disorders lead to elevated uric acid levels. Avan et al. [73] synthesized copper and nitrogen co-doped Ti_3_C_2_T_x_ (Cu@N-Ti_3_C_2_T_x_) and modified it on GCE for the simultaneous detection of adenine and guanine in unnatural sweat samples, with recoveries of 98% to 102%.

Real-time monitoring of chemical and biological substances in biofluids is critical for understanding human health status and implementing precise treatments, while current real-time electrochemical sensors still face several challenges, such as susceptibility to fouling, signal drifting, and short service life. To address the above issues, Liu et al. [74] fabricated an electrochemical microfluidic biosensor by replacing Ti_3_C_2_T_x_-MXene-modified, screen-printed electrodes (SPEs) in a four-layer microfluidic chip, which enabled simultaneous and continuous analysis of three renal function biomarkers, namely urea, UA, and creatinine (Cre), in whole blood (Figure 4b). The detection of UA was based on the direct electrocatalytic oxidation of UA by the MXene/SPE. The detection of urea was based on the further immobilization of urease on the MXene/SPE, which catalyzed the production of NH_3_ from urea, resulting in a change in pH that in turn regulated the electrocatalytic oxidation of UA. The detection of Cre was based on the fact that MXene/SPE adsorbed Cu^2+^ and that Cre selectively formed a complex with Cu^2+^. The installation of a dialysis membrane in the chip avoided additional sample pretreatments. In addition, the sensor employed a ratiometric sensing strategy that adsorbed methylene blue (MB) via MXene as an internal reference signal, thereby greatly reducing signal drift.

Blood glucose (Glu) level is an important indicator of diabetes. In addition, elevated blood UA level is considered one of the best independent predictors of diabetes. Therefore, simultaneous monitoring Glu and UA levels in biofluids is crucial for health management and early diagnosis of diseases. In a study, Cu-TCPP(Fe), a metal–organic framework (MOF) with a large specific surface area, low diffusion resistance, and low conductivity, was integrated with highly conductive Ti_3_C_2_T_x_-MXene. The Cu-TCPP(Fe)/MXene heterostructure was then modified on a paper-based electrode to develop a transient electrochemical sensor based on the superadditive effect mechanism of Cu-TCPP(Fe)/MXene for the simultaneous real-time detection of Glu and UA [75]. The results showed that the strong interfacial interactions between Cu-TCPP(Fe) and Ti_3_C_2_T_x_ greatly improved the electrocatalytic performance and reaction kinetics. The prepared sensor obtained superior performance with unprecedented high sensitivity (Glu: 1.88 aM, UA: 5.80 pM) and wide linear detection range (Glu: 0.001 nM–5 mM, UA: 0.025 nM–5 mM) for Glu and UA, as well as remarkable stability up to 100 days.

Sweat-based electrochemical sensing faces several challenges, such as the short shelf life of conventional electrodes, easy degradation of enzymes, and limited enzyme activity due to the oxygen deficiency in sweat. To address these issues, researchers proposed a stretchable, wearable, and modular biosensor, including a cover layer, a sensor layer, and a sweat uptake layer [76]. The etched pores in the cover layer allowed oxygen to freely enter the enzyme-active layer, ensuring maximized enzyme activity. The unique modular design of sensor layer made it possible to replace the specific sensing electrodes. The oxygen-enriched electrode of the sensor layer consisted of the enzyme, a CNTs-intercalated Ti_3_C_2_T_x_/Prussian blue (PB) porous film, and a superhydrophobic carbon fiber substrate, forming a unique solid–liquid–gas, three-phase interface (Figure 4c). The CNTs/Ti_3_C_2_T_x_/PB composites exhibited excellent conductivity and electrochemical activity, greatly improving the electrochemical performance of the sensor. As a result, the sensor realized the simultaneous detection of Glu and Lac in sweat with high sensitivity (Glu: 35.3 µA/mM·cm^2^, Lac: 11.4 µA/mM·cm^2^) and good repeatability. In addition, a pH-sensing module was incorporated to correct this pH-dependent deviation in the enzyme-based sensor. In another study, a dual-channel electrochemical sensor for real-time monitoring of glucose and lactate in sweat was prepared based on disposable highly integrated sensing (HIS) paper (Figure 4d). To prepare the HIS paper, the paper was first treated with a simple printing process and then folded into a multilayer structure, in which the MXene/methylene blue (Ti_3_C_2_T_x_/MB)-active materials were modified on the working electrodes to facilitate charge migration and biomolecule immobilization. In addition, the 3D sweat diffusion path along the vertical direction of the HIS paper accelerated sweat collection and transport kinetics. The HIS paper-based sensors were demonstrated to be used for simultaneous detection of Glu and Lac with high sensitivity (Glu: 2.4 nA/μM, Lac: 0.49 μA/mM) [77].

#### 4.2.2. Macromolecules

Developing a simple, precise, and controllable method for depositing nanomaterials onto the patterned electrode remains one of the most challenging issues in the fabrication of electrochemical biosensors. To address this issue, Sharifuzzaman et al. [78] developed an innovative electrodeposition strategy for uniformly depositing Ti_3_C_2_T_x_-MXene nanosheets onto a gold dual interdigitated microelectrode (DIDμE). A task-specific ionic liquid, 4-amino-1-(4-formyl-benzyl) pyridinium bromide (AFBPB), was then introduced onto the electrode, which can tightly bind to MXene via electrostatic interaction and π–π stacking, and its aldehydic group also enabled covalent immobilization of biomolecules. Attributed to the homogeneous deposition of conductive MXene and AFBPB, the resultant immunosensor based on MXNSs-AFBPB-modified DIDμE exhibited a 7-fold enhancement in redox current compared to a bare DIDμE. Notably, the immunosensor achieved simultaneous detection of two bladder cancer biomarkers (Apo-A1 and NMP 22) with a wide linear range spanning over five orders of magnitude and low detection limits of 0.3 pg/mL (Apo-A1) and 0.7 pg/mL (NMP 22).

Interleukin-1β (IL-1β) and matrix metalloproteinases-8 (MMP-8) are considered typical salivary biomarkers for early diagnosis and progression monitoring of periodontitis. To meet the clinical need for multiplexed detection, Zhang et al. [79] designed a dual-channel microfluidic electrochemical immunosensor for the simultaneous determination of IL-1β and MMP-8. The integrated system combined a dual-channel chip with two independent SPEs, each of which was modified with iridium oxide (IrOx) nanotubes/Ti_3_C_2_T_x_ nanocomposites. Owing to the synergistic effects of IrOx/Ti_3_C_2_T_x_ nanocomposites to enhance the electron transfer kinetics, the sensor achieved wide detection ranges (IL-1β: 0.1–100 ng/mL, MMP-8: 1–200 ng/mL) and low detection limits (IL-1β: 0.014 ng/mL, MMP-8: 0.13 ng/mL). Additionally, the sensor exhibited remarkable performance in both artificial and clinicopathological saliva analysis, and was promising for point-of-care testing (POCT) for periodontitis diagnosis.

**Figure 5:**
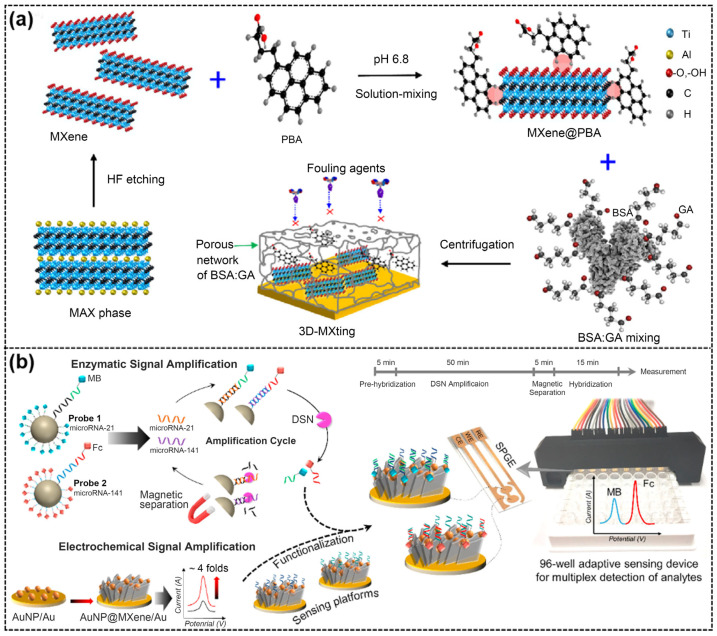
(**a**) Schematic of the preparation process of a 3D-MXting, antifouling nanocomposite. Reproduced with permission from [80]. (**b**) Schematic diagram of the assay procedure for simultaneous detection of microRNA-21 and microRNA-141. Reproduced with permission from [82].

Antifouling coatings are critical for the reliability of biosensors as they prevent non-specific adsorption of interferents. In a recent study, a novel 3D porous antifouling nanocomposite was prepared, which consists of a porous framework of glutaraldehyde (GA) crosslinked with bovine serum albumin (BSA) and 1-pyrenebutyric acid (PBA)-functionalized MXene nanosheets as conductive nanofillers [80]. The oxidation-resistant Ti_3_C_2_T_x_ MXene nanosheets (Al-MXNs) were covalently functionalized with PBA, which facilitated the stable bonding of MXene to the porous framework (Figure 5a). The framework, with its appropriately sized pores, effectively blocked exogenous proteins and minimized interference, while the PBA-functionalized MXene improved the electrochemical lifetime. To test the reliability of the nanocomposite, a multiplexed electrochemical immunosensor was constructed based on 3D-MXting (BSA/Al-MXNs@PBA/GA), antifouling nanocomposite for the simultaneous detection of the inflammatory biomarkers C-reactive protein (CRP) and ferritin, which exhibited excellent detection limits (CRP: 6.2 pg/mL, ferritin: 4.2 pg/mL). In addition, the sensor demonstrated good selectivity and stability in whole serum.

Rapid, accurate, and cost-effective methods for multiplexed detection of infectious disease biomarkers are of great clinical significance. To this end, researchers proposed a multi-channel electrochemical immunosensor with in situ electrodeposition of a MXene/AuNPs composite on SPE [81]. As the AuNPs and MXene nanoflower enlarged the surface area and improved electron transfer efficiency, the sensor achieved highly sensitive detection of three infectious disease biomarkers with wide detection ranges (HBsAg: 0.05–1000 ng/mL, anti-HIV: 0.25–100 ng/mL, anti-TP: 0.35–140 ng/mL), and low detection limits (HBsAg: 0.01 ng/mL, anti-HIV: 0.10 ng/mL, anti-TP: 0.11 ng/mL). Notably, this is the first research that can simultaneously detect three infectious disease biomarkers from a single serum sample.

MicroRNAs are potent clinical biomarkers for early cancer diagnosis. However, sensitive multiplexed detection of microRNAs for accurate cancer diagnosis remains a challenge due to their homologous sequences and low abundance in biofluids. To address these issues, Mohammadniaei and coworkers [82] developed an electrochemical biosensor based on a home-made, screen-printed gold electrode (SPGE) by combining AuNP@MXene electrochemical signal amplification and duplex-specific nuclease (DSN)-based signal amplification strategies (Figure 5b). MXene enhanced the electrochemical signal of the electrode by nearly 4 times, attributed to its large surface area and high charge mobility. For DSN amplification, functionalized magnetic probes with MB- or Fc-labeled, single-stranded DNA (ssDNA) were designed for target-specific cleavage and redox signal generation via DSN amplification. The fabricated biosensor enabled rapid (80 min), attomolar, and simultaneous quantification of two cancer biomarkers, microRNA-21 and microRNA-141. Moreover, this developed strategy, combined with a 96-well adaptive sensing device, enabled the successful analysis of three cancer plasma samples.

#### 4.2.3. Multi-Type Analytes

The emerging MXene-based electrochemical sensors for multiplexed detection in biofluids are no longer limited to a single type of biomarkers but have expanded to simultaneously identify multiple types. These sensors can provide more comprehensive insights for early diagnosis, real-time monitoring, and therapeutic efficacy evaluation assessment of diseases. Furthermore, this capability allows for the integrated diagnosis of multiple diseases through a single analytical platform, thereby enhancing clinical decision-making and personalized healthcare strategies.

Carbon fiber paper (CFP), a 3D carbon material made of interlaced carbon fibers, is considered as an applicable flexible substrate due to its ultra-high conductivity, rich electroactive sites, and good flexibility. In a study, the Huo group reported a flexible electrochemical biosensing platform based on a CFP-Ti_3_C_2_T_x_-MoS_2_-sensing interface for the detection of AA, DA, UA, and microRNA in biofluids [83]. The design of Ti_3_C_2_T_x_-MoS_2_ heterostructure uniformly loaded on CFP facilitated the enrichment of biomolecules, and thus the platform can be used for the simultaneously sensitive detection of AA, DA, and UA. Based on this, capture probes were introduced into the interface by electrodeposition of Au NPs. Combined with the hybridization chain reaction (HCR) and horseradish peroxidase (HRP)-catalyzed amplification, the platform realized the aM level microRNA detection. Notably, the platform also performed well in complex biofluids.

**Figure 6:**
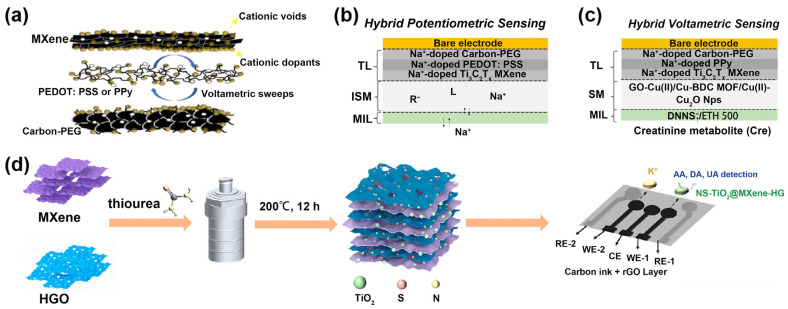
(**a**) Mechanism to dope cationic ions via a carbon-PEG/PEDOT:PSS or PPy/Ti_3_C_2_T_x_ nanosheet. (**b**) Potentiometric sensing mechanism to detect Na^+^ in sweat interference. (**c**) Voltametric sensing mechanism to detect creatinine in sweat interference. Reproduced with permission from [84]. (**d**) Schematic of the preparation process of NS-TiO_2_@MXene-HG and NS-TiO_2_@MXene-HG/rGSPE. Reproduced with permission from [85].

The new generation of textile electrodes (TEs) incorporating capacitive transducers can provide stable signal monitoring in long-term healthcare analysis. In view of this, Kalasin and Sangnuang developed a multiplexed, wearable electrochemical sensor based on sodiated polymers and MXene nanosheet-modified textile electrodes (TEs) for the potentiometric measurement of sodium ion (Na^+^) and the voltametric measurement of Cre in sweat [84]. To fabricate such sensors, sodiated poly(3,4-ethylenedioxythiophene) polystyrenesulfonate (PEDOT:PSS) and polypyrrole (PPy) were inserted between sodiated, nanoporous layer carbon–polyethylene glycol (PEG) and sodiated Ti_3_C_2_T_x_ nanosheets for the potentiometric measurement of Na^+^ and voltametric measurement of creatinine, respectively (Figure 6a–c). This structural design minimized electron transfer loss. In addition, an electrochemical minimizing-interference layer (MIL) was incorporated into the ion-selective membrane (ISM) and creatinine-sensing material (SM), respectively, to minimize the sweat interference. The developed wearable sensors exhibit excellent long-term stability and minimal potential drift in sweat analysis.

Flexible sweat sensors capable of detecting multi-type biomarkers can provide valuable information for understanding personal health. In a study, a novel composite, NS-TiO_2_@MXene-HG, consisting of nitrogen and sulfur co-doped MXene and holey graphene (HG), as well as TiO_2_ nanoparticles grown in situ on the MXene, was synthesized by a facile hydrothermal and then modified on a reduced graphene oxide (rGO) screen-printed electrode (rGSPE) to develop a flexible electrochemical sensor (Figure 6d). Since doped MXene and HG promoted electron transfer and increased active sites, and TiO_2_ enhanced electrocatalytic activity, the NS-TiO_2_@MXene-HG/rGSPE enabled sensitive detection of AA, UA, and DA simultaneously. In addition, a potassium ion-selective, membrane-modified electrode (K^+^-ISM/rGSPE) was fabricated by modifying a K^+^-selective membrane on rGSPE, which realized simultaneous detection of K^+^, AA, UA, and DA in sweat [85].

### 4.3. Drugs

Drug monitoring in biofliuds is essential for facilitating personalized therapy and reducing drug side effects [101]. On the one hand, because individual factors (e.g., age, gender, addictions, liver and kidney function) affect the pharmacokinetics, the actual response and therapeutic efficacy of a drug varies significantly among individuals [4]. Accurate monitoring of drug concentrations in biofluids can assist physicians in developing personalized treatment plans. On the other hand, many therapeutic drugs cause severe adverse effects, such as cardiovascular risks, cerebral risks, gastrointestinal toxicity, hepatotoxicity, and renal injuries [102]. Monitoring the drug concentration in biofluids allows for timely adjustment of drug dosages to avoid the risk of overdose. In addition, the combination of multiple drugs is becoming more common in modern medicine, such as the combination of anticancer drugs and antibiotics in cancer treatment [100], which increases the risk of drug interactions and adverse reactions. Therefore, the simultaneous detection of multiple drugs in biofluids is particularly important, which can provide comprehensive drug information for clinical treatment and improve therapeutic efficacy.

**Figure 7:**
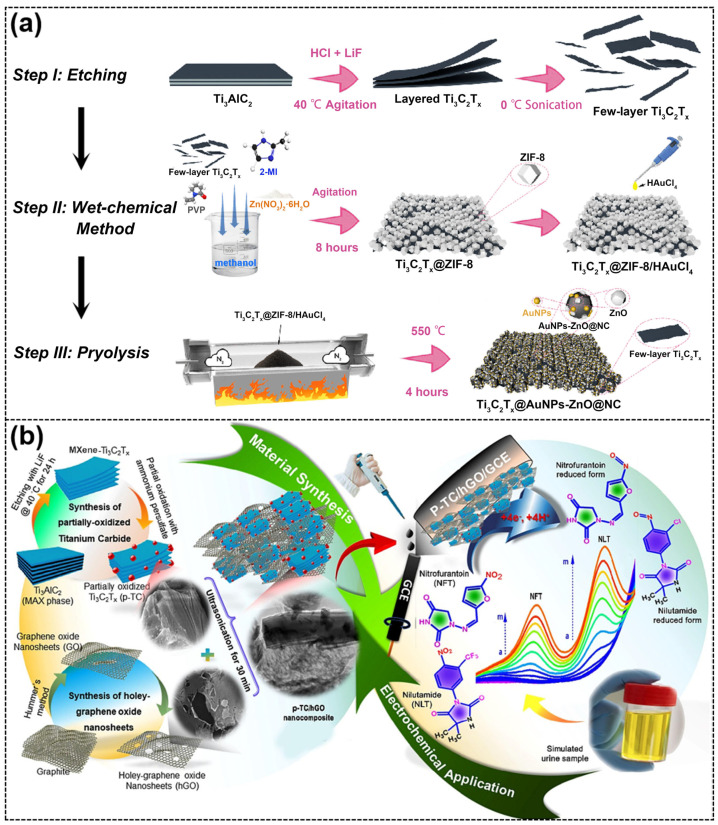
(**a**) Schematic of the preparation procedure of Ti_3_C_2_T_x_@AuNPs-ZnO@NC. Reproduced with permission from [88]. (**b**) Illustration of the synthesis of p-TC/hGO nanocomposite and its application in NFT and NLT simultaneous electrochemical detection. Reproduced with permission from [90].

Acetaminophen (ACOP, also known as paracetamol) is a typical hepatotoxic drug widely used to treat pain and fever. Isoniazid (INZ) is another common hepatotoxic drug mainly used to treat tuberculosis. In a study, a Ti_3_C_2_T_x_-MXene-modified, screen-printed electrode (Ti_3_C_2_T_x_/SPE) was developed for the simultaneous determination of ACOP and INZ [86]. Ti_3_C_2_T_x_/SPE exhibited excellent electrocatalytic activity for both ACOP and INZ compared to bare SPE. During the simultaneous determination of ACOP and INZ, the Ti_3_C_2_T_x_/SPE obtained clearly separated ACOP and INZ oxidation peaks, as well as good analytical performance for the targets with a wide linear range (ACOP: 0.25 to 2000 μM, INZ: 0.1 to 4.6 mM). More importantly, Ti_3_C_2_T_x_/SPE was able to detect ACOP and INZ in the human serum without significant interference. Studies have shown that the combination of paracetamol (PA), theophylline (TP), and caffeine (CA) is utilized for managing childbirth, treating migraine attacks, and avoiding postpartum hemorrhage. In a study, electrochemically active Ti_3_C_2_T_x_ MXene/MWCNT nanocomposites were prepared using microwave and ultrasonication processes and then modified on a SPE for the simultaneous determination of PA, TP, and CA in serum [87]. The prepared Ti_3_C_2_T_x_-MWCNT/SPE demonstrated outstanding performance in detecting PA, TP, and CF in human samples, with good detection limits of 0.23 µM, 0.57 µM, and 0.43 µM, respectively. In another study, Ti_3_C_2_T_x_@Au nanoparticles–ZnO nanoparticles@N-doped carbon (Ti_3_C_2_T_x_@AuNPs-ZnO@NC) was designed for the simultaneous detection of three pharmaceutical molecules—ACOP, DA, and xanthine (XA) [88]—where the detection of DA is useful for the diagnosis of Parkinson’s disease and depression, and the detection of XA facilitates the treatment of asthma. Ti_3_C_2_T_x_@AuNPs-ZnO@NC was prepared by a three-step process of etching, wet chemical treatment, and pyrolysis process (Figure 7a). Since the structural design of the few-layer Ti_3_C_2_T_x_ nanosheets loaded with a large number of AuNPs-ZnO@NC provided electron transport channel for electrochemical reaction, and the NC offered abundant anchoring sites for immobilizing highly active AuNPs, Ti_3_C_2_T_x_@AuNPs-ZnO@NC exhibited excellent electrochemical performance for the simultaneous determination of DA, AC, and XA, with LODs of 41 nM, 59 nM, and 67 nM, respectively. In addition, Ti_3_C_2_T_x_@AuNPs-ZnO@NC could be performed in human blood samples, with recoveries ranging from 93.0% to 108.5%.

Aristolochic acid (AA′) is used in traditional Chinese medicines, but it can cause severe health problems such as urinary epithelial cancer, renal tumors, and renal failure. Roxarsone (ROX) is widely used in poultry farming to gain weight, and its elevated levels can lead to neurodegenerative disorders and several types of cancers in humans. Rajaji et al. [89] prepared an electrochemical sensor based on a laser-induced graphene electrode (LGE) modified with a hybrid of MoS_2_ sphere and sulfur-doped Ti_3_C_2_ MXene (MoS_2_/S-Ti_3_C_2_) for the simultaneous detection of AA′ and ROX. The MoS_2_/S-Ti_3_C_2_ hybrid was synthesized by hydrothermal treatment and calcination. MoS_2_/S-Ti_3_C_2_/LGE exhibited good electrocatalytic performance for AA′ and ROX. In addition, the MoS_2_/S-Ti_3_C_2_/LGE-based sensor demonstrated satisfactory recoveries (97.00–99.00%) for the quantitative detection of AA′ and ROX in human urine and serum samples.

The combination of anticancer drugs and antibiotics is common in cancer chemotherapy, and their overdose can cause severe adverse effects in patients. Nitrofurantoin (NFT) and nilutamide (NLT) are widely used antibiotics and anticancer drugs, respectively. These two drugs have the same electroactive functional group (nitro-group), resulting in signal overlap in electrochemical detection. Therefore, it is crucial to develop novel electrode materials to separate their signals in simultaneous detection. In view of the above, Devi et al. [90] designed a nanocomposite of partially oxidized Ti_3_C_2_T_x_-MXene and holey graphene oxide (p-TC/hGO) for the simultaneous electrochemical detection of NFT and NLT (Figure 7b). The p-TC/hGO nanocomposite was prepared by wet chemical and sonication processes. Since p-TC improved electrical conductivity, hGO increased specific surface area and p-TC flakes and hGO were interconnected to form a conductive network to further facilitate electron transport, p-TC/hGO provided abundant active sites and high electrocatalytic activity for NFT and NLT detection. Compared to the reported literature, the p-TC/hGO/GCE-based sensor demonstrated an outstanding analytical performance in the simultaneous detection of NFT and NLT with low LOD (NFT: 1.2 nM, NLT: 1.9 nM) and ultra-high sensitivity (NFT: 52.8 µA µM^−1^ cm^−2^, NLT: 19.5 µA µM^−1^ cm^−2^). Moreover, the accuracy and real-time application of the sensor were validated in artificial urine samples.

Carbamazepine (CBZ) is an anticonvulsant used to treat epilepsy and trigeminal neuralgia management, and its overdose can cause drowsiness, nausea, leukopenia, and other adverse reactions. Levothyroxine (LT4) is a thyroid hormone replacement drug, and its overdose may lead to risks such as allergy and myocardial infarction. Recently, a highly selective electrochemical sensor based on a MOF-71/V_2_C MXene hydrogel was fabricated by a solvothermal and freeze-drying method for the simultaneous detection of CBZ and LT4 in simulated serum. Combing a porous architecture, large surface area, high catalytic activity, excellent electrical conductivity, and good mechanical flexibility, the MOF-71/V_2_C hydrogel achieved a wide linear detection range (10 nM–100 µM for LT4 and 10 nM–500 µM for CBZ), low detection limits (5.6 nM for LT4 and 6.7 nM for CBZ), excellent selectivity against interferents, and outstanding electrochemical stability. The sensor demonstrated high sensitivity even in complex biological matrices, making it a promising platform for clinical diagnostics and therapeutic drug monitoring [91].

## 5. Conclusions and Outlook

**Figure 8:**
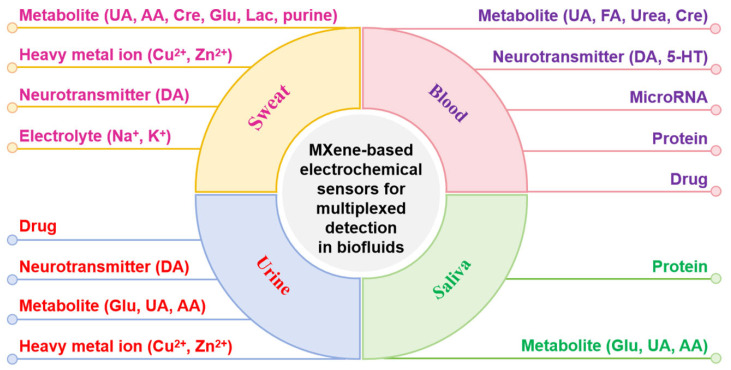
Schematic of biofluids and corresponding targets detected by MXene-based, multiplexed electrochemical sensors.

In recent years, MXene-based electrochemical sensors have been successfully applied to the simultaneous detection of various biofluids, including sweat, urine, blood (whole blood, serum, and plasma), and saliva. These sensors can effectively identify a wide range of targets, such as heavy metal ions (Cu^2+^, Zn^2+^), electrolytes (Na^+^, K^+^), metabolites (UA, AA, FA, Cre, Glu, Lac, urea, purine), neurotransmitters (DA, 5-HT), protein biomarkers, nucleic acids (microRNA), and drugs (Figure 8). Using strategies including the introduction of other materials (metal nanoparticles, metal oxides, carbon nanomaterials, polymers, etc.), heteroatom doping, construction of heterostructures (e.g., MXene/MoS_2_, MXene/MOF), and the construction of 3D porous structures (e.g., MXene-based hydrogels), MXene-based electrode materials with high electrical conductivity, catalytic activity and anti-interference ability were produced. Combined with electrode modification, multi-electrode, or multi-label signal separation strategies, the MXene-based electrochemical sensors have made remarkable advancements in the multiplexed detection of biofluids.

However, MXene-based electrochemical sensors for simultaneous biofluid detection still face some deficiencies or challenges that need further development:

(1) MXene is mainly synthesized through HF etching and in situ HF etching, which results in the formation of -F, -O, and -OH surface groups. These non-uniform terminations are not favorable for the effective immobilization of biorecognition elements. Furthermore, F-terminated MXene exhibit low electrical conductivity [103]. In the future, some strategies can be employed to modulate the surface groups of synthesized MXene to enhance its electrochemical properties [104]. In addition, other fluoride-free synthesis methods for MXene can be explored.
(2) The existing studies focus on the multiplexed detection of sweat, blood, urine, and saliva, while other important biofluids, such as tears and interstitial fluid, have not yet been explored. In the future, detection methods for other biofluids should be developed to extend the application of MXene-based, multiplexed electrochemical sensors.
(3) The current targets mainly cover electrolytes, metabolites, neurotransmitters, proteins, nucleic acids, etc., while the detection of pathogens (e.g., bacteria and viruses) is lacking. In the future, the MXene-based electrochemical sensors for the simultaneous detection of pathogens in biofluids can be further developed.
(4) Real-time monitoring of biofluids is currently focused on SPE-based wearable sensors for sweat detection. However, real-time monitoring of other biofluids, such as interstitial fluid and blood, remains an important area for breakthrough. In the future, wearable microneedle arrays or even implantable sensors are expected to be developed for real-time monitoring of other biofluids [105].
(5) The existing sensors are mostly confined to the simultaneous detection of two to three targets, while the high-throughput detection of more than five targets remains to be achieved. In the future, higher throughput detection can be realized through the fabrication of microelectrode arrays or integration with microfluidic technologies.
(6) The accurate analysis of complex high-throughput data from signal interference in high-throughput electrochemical detection is a great challenge, and the introduction of machine learning techniques provides an effective solution to this problem [106]. In the future, MXene-based, high-throughput electrochemical platforms can be developed in combination with machine learning techniques for more accurate biofluid detection.
(7) Integration of MXene-based, electrochemical multiplexed sensors into clinical POC diagnostic platforms also need to systematically address the regulatory, biocompatibility, and ethical issues [107]. Rigorous quality control and standardized procedures need to be established to ensure the reliability of the sensors in real biofluidic environments, which set high standards for long-term material performance and manufacturing processes. Biocompatibility assessment is crucial, and the potential toxicity, inflammatory response, and long-term in vivo retention effects of MXene-based materials in the target biofluidic environments must be comprehensively examined to ensure their biosafety. In addition, ethical issues related to the protection of user data privacy, the acquisition of informed consent, and the accessibility and fairness of these advanced diagnostic tools also need to be proactively addressed.

In summary, the development of the MXene-based electrochemical sensors for multiplexed detection in biofluid is still on its way. The convergence of advanced manufacturing technologies, artificial intelligence, and biomedical engineering presents unprecedented opportunities for the next-generation of MXene-based, multiplexed electrochemical sensors, and ultimately enables these sensors to truly serve real-time physiological monitoring and personalized healthcare for humans.

## Figures and Tables

**Table 1:** A comparison of different strategies for simultaneous electrochemical detection.

Strategy	Advantages	Disadvantages
Electrode modification	(1) Improves sensitivity and reduces detection limits. (2) Directly distinguishes between targets with similar potentials by altering reaction kinetics. (3) Requires only a single electrode (simple setup).	(1) Limited to electrochemically active analytes. (2) Some materials (e.g., noble metals) are expensive and increase the cost.
Multi-electrode	(1) High robustness and accuracy. (2) Suitable for both electroactive and non-electroactive analytes.	(1) High cost. (2) Requires multi-channel electrochemical analyzers. (3) Fabrication complexity for electrode arrays.
Multi-label	(1) Low cost. (2) Requires only a single electrode. (3) Versatile labels (enzymes, nanoparticles, redox molecules, etc.) and flexible design.	(1) Limited to non-electroactive targets. (2) Require tedious modification steps. (3) Signal interference (cross-reactivity).

**Table 2:** Summary of MXene-based electrochemical sensors for multiplexed detection in biofluids.

Working Electrodes [Ref.]	Signal Separation Strategies	Analytes	Biofluids *	Analytical Methods	Sensitivity	Limit of Detection	Linear Range
Ti_3_C_2_T_x_/MWCNTs/Au [65]	/	Cu^2+^ Zn^2+^	Urine and sweat	SWASV	/	Cu^2+^: 0.1 ppb Zn^2+^: 1.5 ppb	Cu^2+^: 10–600 ppb Zn^2+^: 350–830 ppb
Ti-C-*T*_x_/GCE [66]	Electrode modification	DA UA AA	Urine	DPV	/	AA: 4.64 μM DA: 0.06 μM UA: 0.075 μM	AA: 100–1000 μM DA: 0.5–50 μM UA: 0.5–4 μM & 100–1500 μM
Au-Pd/Ti_3_C_2_T_x_/LSG [67]	Electrode modification	AA DA UA	Urine and sweat	DPV	/	AA: 3 μM DA: 0.13 μM UA: 1.47 μM	AA: 10–1600 μM DA: 12–240 μM UA: 8–100 μM & 200–800 μM
Ti_3_C_2_T_x_/TiO_2_ NWs/GCE [68]	Electrode modification	AA DA UA	Urine	DPV	/	AA: 6.61 μM DA: 0.093 μM UA: 0.038 μM	AA: 300–1800 μM DA: 2–33 μM UA: 2–33 μM
Ti_3_C_2_T_x_/rGO/GCE [69]	Electrode modification	DA UA	Serum	DPV	/	DA: 9.5 nM UA: 0.3 μM	DA: 0.1–100 μM UA: 1–1000 μM
3D rGO-Ti_3_C_2_T_x_/Cu wire [70]	Electrode modification	DA UA	FBS+rat striatum	DPV	DA: 0.74 µA/µM·cm^2^ UA: 2.96 µA/µM·cm^2^; 0.81 µA/µM·cm^2^	DA: 0.061 μM UA: 0.085 μM	DA: 0.5–500 μM UA: 0.5–60 μM; 80–450 μM
Ti_3_C_2_T_x_-PEGDA hydrogel/Au [71]	Electrode modification	DA 5-HT UA	Serum	DPV	/	DA: 2.55 μM 5-HT: 0.83 μM UA: 25.11 μM	DA: 2.5–200 μM 5-HT: 1–100 μM UA: 10–100 μM
AuNP@Ti_3_C_2_T_x_/GCE [72]	Electrode modification	UA FA	Serum	Amperometry (i–t)	UA: 0.530 µA/µM·cm^2^ FA: 0.494 µA/µM·cm^2^	UA: 11.5 nM FA: 6.20 nM	UA: 0.03–1520 μM FA: 0.02–3580 μM
Cu@N-Ti_3_C_2_T_x_/GCE [73]	/	Adenine Guanine	Artificial sweat	DPV	/	adenine: 0.01 μM guanine: 0.02 μM	0.1–10 μM
Urease-MB/Ti_3_C_2_T_x_/SPE for Urea and UA; Ti_3_C_2_T_x_/SPE for Cre [74]	Electrode modification and multi-electrode	Urea UA Cre	Whole blood	SWV	/	Urea: 0.02 mM UA: 5 μM Cre: 1.2 μM	Urea: 0.1–3 mM UA: 30–500 μM Cre: 10–400 μM
GOx(UOx)/Cu-TCPP(Fe)/Ti_3_C_2_T_x_/paper-based electrode [75]	Multi-electrode	Glu UA	Artificial sweat, urine, and saliva	CV	/	Glu: 1.88 aM UA: 5.80 pM	Glu: 0.001 nM–5 mM UA: 0.025 nM–5 mM
GOx(LOx)/CNTs/Ti_3_C_2_T_x_/PB/CFMs [76]	Multi-electrode	Glu Lac	Sweat	CA	Glu: 35.3 µA/mM·cm^2^ Lac: 11.4 µA/mM·cm^2^	Glu: 0.33 μM Lac: 0.67 μM	Glu: 10–1500 μM Lac: 0–22 mM
GO_X_(LO_X_)/MB/Ti_3_C_2_T_x_/SPCE [77]	Multi-electrode	Glu Lac	Sweat	CA & DPV	Glu: 2.4 nA/μM Lac: 0.49 μA/mM	Glu: 17.05 μM Lac: 3.73 μM	Glu: 0.08–1.25 mM Lac: 0.3–20.3 mM
DIDμEs/MXNSs-AFBPB [78]	Multi-electrode	Apo-A1 NMP 22	Urine	DPV	/	Apo-A1: 0.3 pg/mL NMP 22: 0.7 pg/mL	0.1 pg/mL–50 ng/mL
IrO_x_/Ti_3_C_2_T_x_/SPE [79]	Multi-electrode	IL-1β MMP-8	Artificial saliva and clinicopathological saliva	DPV	/	IL-1β: 0.014 ng/mL MMP-8: 0.13 ng/ mL	IL-1β: 0.1–100 ng/mL MMP-8: 1–200 ng/mL
3D-MXting/Au [80]	Multi-electrode	CRP Ferritin	Serum	CV	/	CRP: 6.2 pg/mL Ferritin: 4.2 pg/mL	0.01–100 ng/mL
AuNPs and Ti_3_C_2_T_x_-SPE [81]	Multi-electrode	HBsAg Anti-HIV Anti-TP	Serum	DPV	/	HBsAg: 0.01 ng/mL Anti-HIV: 0.10 ng/mL Anti-TP: 0.11 ng/mL	HBsAg: 0.05–1000 ng/mL Anti-HIV: 0.25–100 ng/mL Anti-TP: 0.35–140 ng/mL
AuNP@Ti_3_C_2_T_x_/SPGE [82]	Multi-electrode and multi-label	MicroRNA-21 MicroRNA-141	Plasma	DPV	/	microRNA-21: 204 aM microRNA-141: 138 aM	500 aM–50 nM
CFP-Ti_3_C_2_T_x_-MoS_2_ for AA, DA and UA; CFP-Ti_3_C_2_T_x_-MoS_2_-AuNPs for microRNA [83]	Electrode modification and multi-electrode	AA DA UA MicroRNA	Urine (AA, DA, UA) and serum (microRNA)	DPV	/	AA: 0.89 μM DA: 0.23 μM UA: 0.35 μM MicroRNA: 3.16 aM	AA: 10–1000 μM DA: 0.5–200 μM UA: 0.5–150 μM MicroRNA: 0.1 fM–10 fM; 10 fM to 10 nM
TE/carbon-PEG/PEDOT:PSS/Ti_3_C_2_T_x_/ISM/MIL for Na^+^; TE/carbon-PEG/PPy/Ti_3_C_2_T_x_/SM/MIL for Cre [84]	Multi-electrode	Na^+^ Cre	Sweat	Potentiometry DPV	Na^+^: –58.9 mV/dec Cre: 0.014 ± 0.001 μA/μM	Na^+^: 10^–6.2^ M Cre: 0.12 μM	Na^+^: 10^–6^–10^–1^ M Cre: 0.6–2800 μM
NS-TiO_2_@MXene-HG/rGSPE for AA, DA and UA; ISM/rGSPE for K^+^ [85]	Electrode modification and multi-electrode	AA DA UA K^+^	Sweat	i–t OCPT	AA: 20.78 µA/µM·cm^2^ DA: 32.78 µA/µM·cm^2^ / /	AA: 0.025 μM DA: 0.1 μM UA: 0.14 μM /	AA: 0.1–2200 μM DA: 0.25–100 μM; 100–400 μM UA: 0.25–100 μM; 100–225 μM K^+^: 0.19–24 mM; 24–125 mM
Ti_3_C_2_T_x_/SPE [86]	/	ACOP INZ	Serum	DPV	/	ACOP: 0.048 μM INZ: 0.064 mM	ACOP: 0.25–2000 μM INZ: 0.1–4.6 mM
Ti_3_C_2_T_x_-MWCNT/SPE [87]	Electrode modification	PA TP CF	Serum	DPV	PA: 2.194 µA/µM·cm^2^ TP: 2.179 µA/µM·cm^2^ CF: 5.035 µA/µM·cm^2^	PA: 0.23 µM TP: 0.57 µM CF: 0.43 µM	PA: 1.0–90.1 µM TP: 2.0–62.0 µM CF: 2.0–90.9 µM
Ti_3_C_2_T_x_@AuNPs-ZnO@NC/GCE [88]	/	DA ACOP XA	Blood	DPV	/	DA: 0.041 μM AC: 0.059 μM XA: 0.067 μM	DA: 3–200 μM AC: 15–500 μM XA: 8–350 μM
MoS_2_/S-Ti_3_C_2_/LGE [89]	/	AA’ ROX	Urine and serum	DPV	AA’: 69.955 µA/µM·cm^2^; 32.488 µA/µM·cm^2^ ROX: 56.972 µA/µM·cm^2^; 19.688 µA/µM·cm^2^	AA’: 1.65 nM ROX: 2.31 nM	0.01–875.01 μM
p-TC/hGO/GCE [90]	Electrode modification	NFT NLT	Artificial urine	DPV	NFT: 52.8 µA/µM·cm^2^ NLT: 19.5 µA/µM·cm^2^	NFT: 1.2 nM NLT: 1.9 nM	NFT: 0.05–135 μM NLT: 0.05–158 μM
MOF-71/V_2_C MXene–hydrogel [91]	/	LT4 CBZ	Simulated serum	DPV	/	LT4: 5.6 nM CBZ: 6.7 nM	LT4: 10 nM–100 μM CBZ: 10 nM–500 μM

Abbreviation: MWCNTs: multiwalled carbon nanotubes; GCE: glassy carbon electrode; DA: dopamine; UA: uric acid; AA: ascorbic acid; LSG: laser-scribed graphene; TiO_2_ NWs: TiO_2_ nanowires; rGO: reduced graphene oxide; FBS: fetal bovine serum; PEGDA: poly(ethylene glycol) diacrylate; 5-HT: 5-hydroxytryptamine; FA: folic acid; Cu@N-Ti_3_C_2_T_x_: copper and nitrogen co-doped Ti_3_C_2_T_x_; MB: methylene blue; SPE: screen-printed electrode; Cre: creatinine; GOx: glucose oxidase; UOx: uric acid oxidase; Glu: glucose; LOx: lactate oxidase; PB: Prussian blue; CFMs: carbon fiber membranes; Lac: lactate; SPCE: screen-printed carbon electrode; DIDμEs: dual interdigitated microelectrodes; MXNSs: Ti_3_C_2_T_x_-MXene nanosheets; AFBPB: 4-amino-1-(4-formyl-benzyl) pyridinium bromide; IL-1β: interleukin-1β; MMP-8: matrix metalloproteinase-8; 3D-MXting: BSA/Al-Ti_3_C_2_T_x_@1-pyrenebutyric acid/glutaraldehyde; CRP: C-reactive protein; SPGE: screen-printed gold electrodes; CFP: carbon fiber paper; TE: textile electrode; PEG: polyethylene glycol; PEDOT:PSS: poly(3,4-ethylenedioxythiophene) polystyrenesulfonate; ISM: ion-selective membrane; MIL: minimizing-interference layer; PPy: polypyrrole; SM: sensing material; NS-TiO_2_@MXene-HG: nitrogen and sulfur co-doped holey graphene and MXene, with in situ-grown TiO_2_ nanoparticles on the MXene; rGSPE: reduced graphene oxide-modified screen-printed electrode; OCPT: open circuit potential test; ACOP: acetaminophen; INZ: isoniazid; PA: paracetamol (acetaminophen); TP: theophylline; CF: caffeine; NC: N-doped carbon; LGE: laser-induced graphene electrode; XA: xanthine; AA’: aristolochic acid; ROX: roxarsone; p-TC/hGO: nanocomposite of holey graphene oxide with partially oxidized titanium carbide; NFT: nitrofurantoin; NLT: nilutamide; MOF: metal–organic framework; LT4: levothyroxine; CBZ: carbamazepine. *: Unless otherwise noted, biofluids in the table refer to those from humans.
